# Editorial: Molecular characteristics and personalized treatment for pediatric brain tumors

**DOI:** 10.3389/fonc.2022.1114280

**Published:** 2022-12-29

**Authors:** Weijun Feng, Daisuke Kawauchi, Feng Wan

**Affiliations:** ^1^ Institute of Pediatrics, Children’s Hospital of Fudan University, Shanghai Key Laboratory of Medical Epigenetics, International Co-Laboratory of Medical Epigenetics and Metabolism, Ministry of Science and Technology, Institutes of Biomedical Sciences, Fudan University, Shanghai, China; ^2^ Department of Biochemistry and Cellular Biology, National Center of Neurology and Psychiatry (NCNP), National Institute of Neuroscience, Tokyo, Japan; ^3^ Department of Neurosurgery, Guangdong Provincial People’s Hospital, Guangdong Academy of Medical Sciences, Guangzhou, China

**Keywords:** intracranial teratoma, diffuse leptomeningeal glioneuronal tumors, posterior fossa group A ependymoma, En bloc resection, CAR T cell immunotherapy

Pediatric brain tumors (PBTs) are the most common solid childhood cancers and the second most common type of childhood cancers, exceeded in incidence only by leukemia. Currently, high grade PBTs have the highest mortality rate among all childhood cancers (1). Despite advances and refinements in the standard clinical treatment such as neurosurgical techniques, radiation, and chemotherapy, improved outcomes in malignant brain tumors still remain limited (2). New treatment approaches such as immunotherapy are now emerging for treating some refractory PBTs, while challenged by the need for more intimately understanding of the unique interactions between brain tumor cells and immune system (3).

Over the past decade, profound understandings of the molecular and epigenetic landscape of pediatric brain tumors have allowed for improved classification, identification of previously unrecognized entities, and superior risk stratification (2, 4, 5). This has also resulted in molecularly targeted therapy that is beginning to be incorporated into the management of a significant proportion of low-grade glioma, and a small proportion of high-grade glioma (6). Thus, current care for pediatric brain tumors is moving toward more molecularly-based and personalized treatment. For example, the present state-of-the-art molecular diagnosis is often integrated into pre-operative radiology and intra-operative rapid detection for surgical decision and tailored tumor resection.

The current Research Topic focuses on the progress on novel discoveries of PBT biology, molecular and clinical diagnosis, and precision personalized treatments. Within this issue, we have collected two research articles, two review articles and one case report related with the theme.

Intracranial teratoma is a rare tumor occurring in the central nervous system and often classified into subgroups pathologically. Due to its rarity, the molecular profile of intracranial teratoma have remained elusive. Here, Zhang et al. collected 46 patients bearing this type of tumor and 22 of these were analyzed by whole-exome sequencing, resulting in discovery of recurrent somatic mutations and copy number variations, some of which have been known as a cancer-related mutation. By integration of the molecular data with histological analysis, they found that deletions of *SETDB2* and *IL2* occur in an immature teratoma-specific manner. Furthermore, survival analysis revealed that 2 out of 3 patients who died after tumor relapse had *TP53* mutations, newly suggesting that *TP53* mutation could be a prognostic factor for intracranial teratoma. In the future, the authors hope to establish a mouse model using patient-derived xenografts to identify oncogenic signals that are functional and to conduct preclinical studies based on those studies.

Jiang et al. focused their study on another rare pediatric brain tumor, diffuse leptomeningeal glioneuronal tumor (DLGNT). The clinical progression, pathological characteristics, and MRI findings of 145 patients were summarized based on literature review. DLGNT presents specific radiological features including hydrocephalus, diffuse enhancement of meninges and multiple nodules. These findings highlight the need to integrate clinical and imaging results to improve the early and accurate diagnosis of DLGNT.

Ependymoma is the third most common pediatric primary brain tumor, with its most aggressive subtype being posterior fossa group A (PFA). Zhou et al. report the first case of pediatric PFA ependymoma with extraneural i.e., osseous, and pulmonary metastases. The primary tumor was characterized with elevated chromosome X open reading frame 67 (CXorf67)/EZHIP and the resulting loss of the H3K27me3 mark, which are typical features of PFA. Genomic analysis of metastatic tumors revealed deletion of the *CDKNA2A/B* locus and mutation of *CDK12*. Moreover, Zhou et al. systematically reviewed the literature to summarize the potential mechanism of extra-neural metastasis of ependymoma, despite requirement of functional validation in the future.

Surgical resection remains as the most effective and primary treatment of most PBTs. Children are less sustained with blood loss during the surgery. To perform a more extensive tumor resection while minimizing surgical morbidity, Cao et al. emphasized en bloc technique, which is less frequently reported in brain tumor surgery. In their retrospective study, the en bloc resection group had a shorter intensive care unit and hospital stay, less surgical blood loss and lower transfusion rate, as well as reduced complication rate. An en bloc resection might not always be successfully implemented during brain tumor surgery, depending on the many tumor growth features as mentioned in the manuscript. While the en bloc concept is advocated whenever possible, especially in pediatric patients for a better control of vascular supply to the tumor.

Current treatment of pediatric brain tumor includes surgery, chemotherapy and radiotherapy. Both the efficacy and long-term neurological side effects need to be improved. Immunotherapy with genetically engineered T cells with chimeric antigen receptors (CARs), have provided new hope for patients as a promising therapeutic avenue. In their review article, Rao et al. illustrate recent advances in clinical and pre-clinical development of CAR-T cell immunotherapy in pediatric brain tumors including diffuse midline gliomas and pediatric high-grade gliomas. Multiple clinical trials of CAR-T cell therapy in PBTs are ongoing, with targets such as IL13Rα2, GD2, HER2, EGFR and B7-H3. CAR-T cell therapy in PBTs faces similar challenges as adult brain tumors, like the heterogenous tumor microenvironment (TME) and delivery of CAR-T cells across the blood brain barrier (BBB). Rao et al. summarize the current understanding of immunosuppression in the pediatric brain tumor TME and potential overcoming approaches as well. It is now well known that PBTs have distinct molecular characterizations compared to adult brain tumors, for instance, whether PBT-specific histone H3.3 and H3.1 mutations could serve as new antigen targets for CAR-T cell therapy.

In summary, our Research Topic covered the molecular characterization of a few rare pediatric brain tumors and personalized treatment including an improved tumor resection concept and CAR-T cell immunotherapy. Hopefully, our topic can contribute to further understanding and better treatment of the pediatric brain tumors.

## Author contributions

All authors listed have made a substantial, direct, and intellectual contribution to the work and approved it for publication.

## References

[1] Ostrom QT Cioffi G Gittleman H Patil N Waite K Kruchko C. CBTRUS statistical report: Primary brain and other central nervous system tumors diagnosed in the united states in 2012-2016. Neuro Oncol (2019) 21(Suppl 5):v1–100. doi: 10.1093/neuonc/noz150 31675094 PMC6823730

[2] Cohen AR. Brain tumors in children. N Engl J Med (2022) 386(20):1922–31. doi: 10.1056/NEJMra2116344 35584157

[3] Wang SS Bandopadhayay P Jenkins MR. Towards immunotherapy for pediatric brain tumors. Trends Immunol (2019) 40(8):748–61. doi: 10.1016/j.it.2019.05.009 31229353

[4] Louis DN Perry A Wesseling P Brat DJ Cree IA Figarella-Branger D. The 2021 WHO classification of tumors of the central nervous system: A summary. Neuro Oncol (2021) 23(8):1231–51. doi: 10.1093/neuonc/noab106 PMC8328013 34185076

[5] Petralia F Tignor N Reva B Koptyra M Chowdhury S Rykunov D. Integrated proteogenomic characterization across major histological types of pediatric brain cancer. Cell (2020) 183(7):1962–85. doi: 10.1016/j.cell.2020.10.044 PMC8143193 33242424

[6] Nör C Ramaswamy V. Piecing together the pediatric brain tumor puzzle. Trends Genet (2021) 37(3):204–6. doi: 10.1016/j.tig.2021.01.001 33455817

